# Does sex matter? Association of fetal sex and parental age with pregnancy outcomes in Taiwan: a cohort study

**DOI:** 10.1186/s12884-020-03039-y

**Published:** 2020-06-08

**Authors:** Tsung Yu, Ta-Sheng Chen, Fu-Wen Liang, Pao-Lin Kuo

**Affiliations:** 1 Department of Public Health, National Cheng Kung University Hospital, College of Medicine, National Cheng Kung University, Tainan, Taiwan; 2 Department of Obstetrics and Gynecology, National Cheng Kung University Hospital, College of Medicine, National Cheng Kung University, Tainan, Taiwan; 3 Department of Public Health, College of Health Sciences, Kaohsiung Medical University, Kaohsiung, Taiwan

**Keywords:** Fetal sex, Parental age, Gestational hypertension, Preeclampsia, Preterm delivery

## Abstract

**Background:**

Worldwide several studies have examined the associations of fetal sex, paternal age and maternal age with pregnancy outcomes, with the evidence regarding paternal age being less consistent. Although in Taiwan we keep good records on birth certificates, these issues have been seldom researched. Our objective was to assess the association of fetal sex and parental age with gestational hypertension/preeclampsia, eclampsia and preterm delivery in the Taiwanese population.

**Methods:**

We conducted a nationwide study and included 1,347,672 live births born between 2004 and 2011 in Taiwan. Gestational hypertension/preeclampsia and eclampsia were ascertained based on the International Classification of Diseases codes; preterm delivery (< 37 weeks) was defined according to the gestational age documented by healthcare providers. We implemented logistic regression models with covariates adjusted to assess the association of fetal sex and parental age with pregnancy outcomes.

**Results:**

The prevalence was 2.27% for gestational hypertension/preeclampsia, 0.07% for eclampsia and 6.88% for preterm delivery. After considering other parent’s age and covariates, we observed a significantly stepped increase in the risk of both gestational hypertension/preeclampsia and preterm delivery as paternal and maternal age increased. For example, fathers aged ≥50 years were associated with a significantly higher risk of gestational hypertension/preeclampsia (odds ratio [OR]: 1.60, 95% CI: 1.39, 1.84) and preterm delivery (OR: 1.38, 95% CI: 1.27, 1.51) than fathers aged 25–29 years. Analysis on fetal sex showed that relatively more female births were linked to gestational hypertension/preeclampsia and more male births linked to preterm delivery, compared to the whole population.

**Conclusions:**

We found both paternal and maternal age, as well as fetal sex, were associated with the risk of pregnancy outcomes. Some findings on fetal sex contradicted with previous research using non-Asian samples, suggesting that ethnicity may play a role in the association of fetal sex and pregnancy outcomes. Besides, there is a need to counsel couples who are planning their family to be aware of the influence of both advanced maternal and paternal age on their pregnancy outcomes.

## Background

Sex is an important aspect of perinatal medicine. For example, the sex of the fetus is associated with adverse pregnancy outcomes. Studies suggest that a male fetus is related to a higher risk of miscarriage, stillbirth and infant death than a female fetus [1, 2]. Also, several studies have found that pregnant women expecting a male fetus are at higher risk of gestational diabetes, gestational hypertension/preeclampsia, and preterm delivery, although other studies suggest conflicting findings [3–7]. These pregnancy complications strongly relate to the dysfunction of human placenta. Previous research showed that the gene expression for growth and maintenance of pregnancy is fetal sex-based [8] and now scientists deem human placenta a sexually dimorphic organ [9]. Thereby, some studies found that the effect of adverse maternal uterine environment on the fetus is sex-specific, suggesting the importance of considering fetal sex when we study the etiology of pregnancy complications [10–12].

Besides fetal sex, the sex of the parents matters as well. Of particular relevance is the influence of advanced paternal age and maternal age on adverse pregnancy outcomes. Advanced maternal age increases the risk of spontaneous abortion, ectopic pregnancy, congenital malformation, perinatal mortality, and maternal conditions such as gestational diabetes and gestational hypertension/preeclampsia [13]. Advanced paternal age, while less influential on pregnancy outcomes than maternal age, is related to chromosomal and non-chromosomal birth defects, autism spectrum disorder, and childhood cancer [14]. Over the years across developed countries, couples are delaying their childbearing age along with increased educational level, later marriage, and advances in assisted reproductive technology [15, 16]. In Taiwan, postponement of parenthood is particularly evident. The mean age of all mothers at birth was 25.4 years in 1975 and for fathers, the mean age was 30.6 years [17]. Recent statistics showed that in 2018 the mean age for mothers and fathers was 32.0 years and 34.5 years, respectively [17]. It is therefore important to assess the impact of delaying parenthood at a population level and understand its public health implications.

We focused our attention mainly on two types of adverse pregnancy outcomes that relate to maternal and perinatal morbidity and mortality; that is, hypertensive disorders of pregnancy (gestational hypertension, preeclampsia and eclampsia) and preterm delivery. Hypertensive disorders induced by pregnancy may lead to stillbirth, infant death, and maternal death [18]. It also increases women’s long-term risk of developing chronic hypertension, cardiovascular disease, stroke and venous thromboembolism [19]. Preterm delivery affects about 10% of newborns globally [20]. Preterm delivery is associated with many adverse perinatal outcomes, such as respiratory distress syndrome, sepsis, neurological impairment, and bronchopulmonary dysplasia, and it also increases perinatal mortality [21]. Moreover, preterm delivery incurs significant cost to the healthcare system and the psychological burden on families [22].

The main purpose of this research was to elucidate how fetal sex, maternal and paternal age are associated with gestational hypertension/preeclampsia, eclampsia and preterm delivery in the Taiwanese population. Besides, given that placenta is a sexually dimorphic organ, the prenatal stress induced by advanced parental age may interact with fetal sex. Specifically, we aimed to (1) quantify the effect of fetal sex on the risk of gestational hypertension/preeclampsia, eclampsia and preterm delivery, (2) quantify the effect of paternal age and maternal age on such outcomes, and (3) explore whether the effect of paternal age and maternal age varies according to fetal sex.

## Methods

### Data source

The current study was a nationwide, population-based, retrospective cohort study conducted in Taiwan, using live birth data collected between January 1, 2004 and December 31, 2011. We obtained data from the Taiwan Maternal and Child Health Database, which consisted of data from Birth Reports, Death Registrations, and medical claims information from the National Health Insurance program [23]. In Taiwan, the law requires that healthcare providers report births to the government, and the information to report includes sociodemographics, prenatal care, risk factors in pregnancy, route of delivery and newborn characteristics at birth, e.g., sex, birthweight and gestational age. To obtain clinical visit data of both parents and children, the Taiwan Maternal and Child Health Database was also linked through encrypted personal identifiers to the claims database from the National Health Insurance program. It is a social health insurance plan instituted in Taiwan and has a population coverage of 99% [24]. We used the inpatient and outpatient medical claims data for assessing maternal medical conditions during pregnancy. The details about the setup of the Taiwan Maternal and Child Health Database and research reports using this database can be found elsewhere [25, 26].

The study protocol was approved by the Institutional Review Board at the National Cheng Kung University in Tainan, Taiwan (Number: A-ER-108-245). Datasets were obtained from the Health and Welfare Data Science Center of Taiwan and de-identified when investigators were performing data analysis.

### Demographic variables and covariates

The main exposure of our study was paternal age, maternal age, and fetal sex. We calculated the paternal and maternal age on the child’s birthday using the date of birth of the parents. After a discussion within our team, we included two covariates in the adjusted models for which we had complete information. These two covariates were maternal nationality (Taiwanese vs. immigrant) and family income level. Family income data were collected by the Taiwan’s National Health Insurance Program and based on the monthly salary of the parents. Income data were categorized into quartiles.

### Outcomes

For the current study, pregnancy outcomes of interest were preterm delivery, gestational hypertension/preeclampsia, and eclampsia. Gestational age was used to define a preterm delivery and the information was documented on the Birth Reporting form by healthcare providers. We defined preterm delivery to be gestational age < 37 weeks.

We identified a maternal diagnosis of gestational hypertension, preeclampsia or eclampsia using inpatient and outpatient medical claims data. We used the International Classification of Diseases codes (ICD-9-CM): 642.* for gestational hypertension/preeclampsia and 642.6* for eclampsia. We decided to make gestational hypertension/preeclampsia a composite outcome because our database identified mostly women with severe gestational hypertension while mild cases might not receive the diagnostic codes. To increase the specificity for diagnosis, mothers classified as having gestational hypertension/preeclampsia had at least one hospital admission record or at least two outpatient visit records indicated for the condition.

### Statistical analysis

Initially, there were 1,586,557 live birth records in the database from 2004 to 2011. We excluded individuals with missing data on maternal or paternal age, and we further restricted our study population to those with a paternal age of 10 to 90 years and a maternal age of 10 to 60 years, to exclude improbable ages. We excluded multiple pregnancies and included only singleton births (see Supplemental Figure 1 for study flow). We conducted statistical analyses using SAS version 9.3 (SAS Institute, Cary, NC, USA).

We constructed logistic regression models to investigate the association of fetal sex, paternal age and maternal age with pregnancy outcomes, taking into account the covariates. It is possible a woman may have more than one pregnancy during the study time frame (2004–2011); to account for the correlated nature of repeated pregnancies within the same women during the study time frame, we used generalized estimating equation with an exchangeable correlation structure as the analytical approach.

In the regression analysis, paternal and maternal age was modeled using dummy variables since their association with pregnancy outcomes may be non-linear. Paternal age was categorized into < 25 years, 25 to 29 years (reference group), 30 to 34 years, 35 to 39 years, 40 to 44 years, 45 to 49 years, and ≥ 50 years. Maternal age was categorized into < 20 years, 20 to 24 years, 25 to 29 years (reference group), 30 to 34 years, 35 to 39 years, and ≥ 40 years. For every outcome, we started with constructing unadjusted models; 25 to 29 years was used as the reference group for age. We obtained the odds ratios and 95% confidence intervals that quantified the influence of fetal sex and each age group on the outcomes.

Next, we constructed adjusted logistic regression models, for which we included variables of fetal sex, paternal age category, maternal age category, maternal nationality and income level. These multivariable models were constructed mainly to isolate the independent influence of fetal sex, paternal age and maternal age on the outcomes.

Finally, we conducted stratified analyses by fetal sex to explore whether the influence of paternal age or maternal age on these pregnancy outcomes varied according to fetal sex. We also computed the sex ratios (male/female) stratified by paternal age or maternal age for the entire population, and for those births with gestational hypertension/preeclampsia and for preterm deliveries.

## Results

We identified 1,586,557 live birth records between 2004 and 2011 in the Taiwan Maternal and Child Health Database. We then excluded multiple pregnancies (such as twins and triplets) and births with missing data on maternal or paternal age and births with improbable maternal or paternal age, leaving 1,347,672 live birth records for the final analysis (see Supplemental Figure 1).

The distribution of selected characteristics is shown in Table 1. Age group 30–34 years accounted for most of all fathers (39%) and age group 25–29 years accounted for most of all mothers (36%). The Pearson correlation coefficient between paternal and maternal age was 0.64. The prevalence of gestational hypertension/preeclampsia, eclampsia and preterm delivery was 2.27, 0.07, and 6.88%, respectively. Male births accounted for 52% of all live births and the sex ratio was 1.10.

**Table 1:** Characteristics of live births

Characteristics	***N*** = 1,347,672
**Paternal age, y**	
< 25, *n (%)*	75,119 (6)
*25–29, n (%)*	328,463 (24)
*30–34, n (%)*	520,954 (39)
*35–39, n (%)*	301,149 (22)
*40–44, n (%)*	93,068 (7)
*45–49, n (%)*	21,974 (2)
*≥ 50, n (%)*	6945 (1)
**Maternal age, y**	
< 20, *n (%)*	23,195 (2)
*20–24, n (%)*	185,607 (14)
*25–29, n (%)*	487,567 (36)
*30–34, n (%)*	474,148 (35)
*35–39, n (%)*	156,352 (12)
*≥ 40, n (%)*	20,803 (2)
**Maternal nationality**	
*Immigrant, n (%)*	88,025 (7)
*Taiwanese, n (%)*	1,259,647 (93)
**Income**	
*Dependents or 1st quartile, n (%)*	328,224 (24)
*2nd quartile, n (%)*	340,771 (25)
*3rd quartile, n (%)*	339,904 (25)
*4th quartile, n (%)*	338,773 (25)
**Route of delivery**	
*Vaginal, n (%)*	892,366 (66)
*Cesarean, n (%)*	455,306 (34)
**Gestational hypertension or preeclampsia,*n (%)***	30,526 (2)
**Eclampsia,*n (%)***	944 (0)
**Infant sex**	
*Boy, n (%)*	704,523 (52)
*Girl, n (%)*	643,149 (48)
*Sex ratio*	1.10
**Mean gestational age,*wk (SD)***	38 (2)
**Preterm birth,*n (%)***	92,663 (7)
**Mean birthweight,*g (SD)***	3112 (426)

*SD* standard deviation

**Fig. 1:**
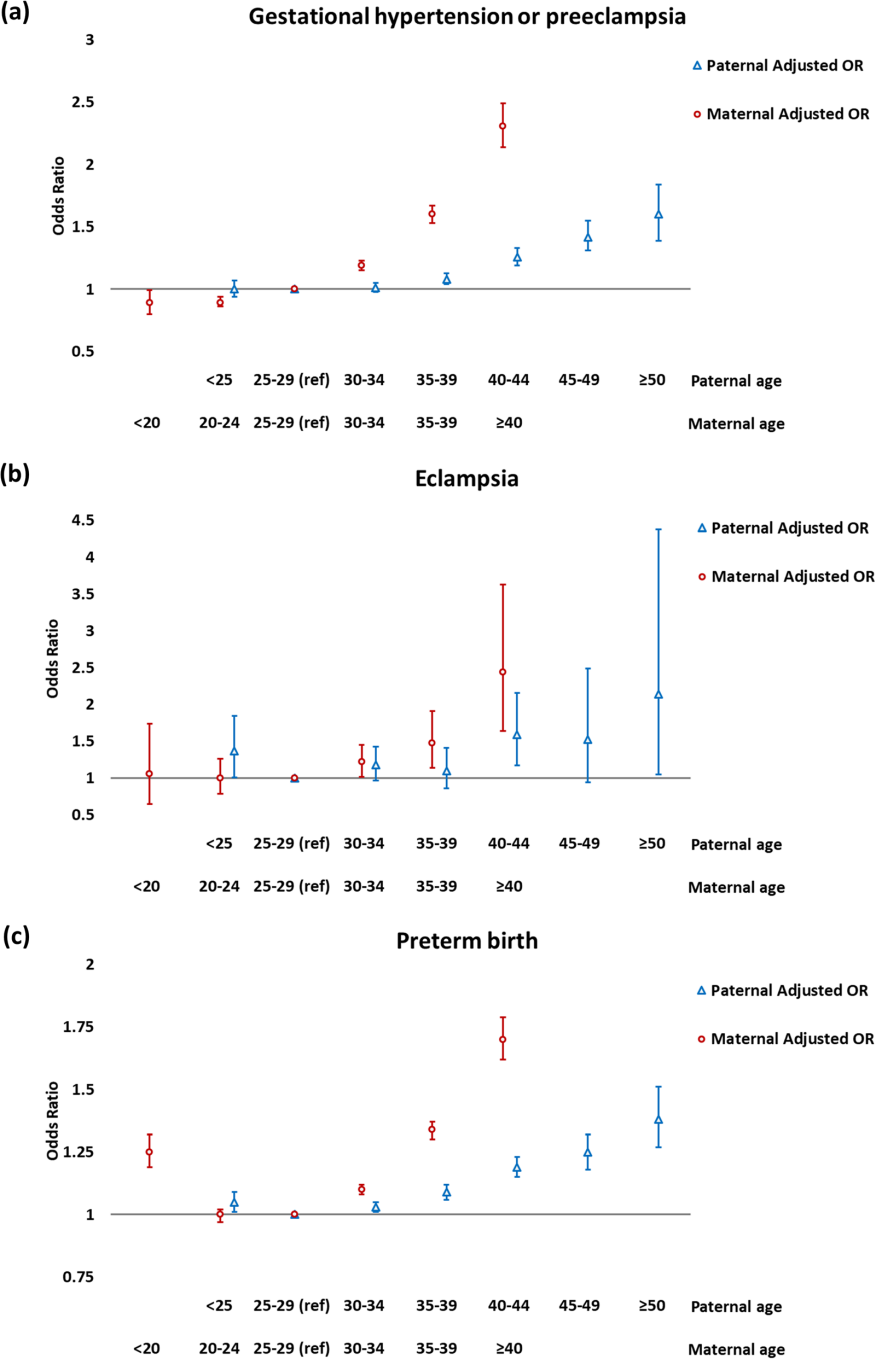
Adjusted odds ratios (ORs) for pregnancy outcomes by paternal and maternal age categories

Estimates of the association of paternal and maternal age with pregnancy outcomes (odds ratio [OR] and 95% confidence interval [CI]) are shown in Table 2; we also present the adjusted results in the Fig. 1. In the unadjusted analysis, we found that both older paternal age and older maternal age were associated with worse pregnancy outcomes, and observed a stronger effect for maternal age. For instance, compared with mothers aged 25–29, the ORs for gestational hypertension/preeclampsia were 1.18 (95% CI: 1.15, 1.21) for mothers aged 30–34, 1.69 (95% CI: 1.63, 1.75) for 35–39 and 2.76 (95% CI: 2.58, 2.94) for ≥40. After adjustment for the other parent’s age and covariates in the multivariable analysis, these effects (ORs) for paternal and maternal age were attenuated. Mothers carrying a male fetus were less likely to have gestational hypertension/preeclampsia.

**Table 2:** Crude and adjusted odds ratios for outcomes associated with paternal age, maternal age and fetal sex (total live births = 1,347,672)

	Gestational hypertension or preeclampsia (***n*** = 30,526)		Eclampsia (***n*** = 944)		Preterm birth (***n*** = 92,663)	
	Crude OR (95% CI)	Adjusted OR (95% CI)^**a**^	Crude OR (95% CI)	Adjusted OR (95% CI)^**a**^	Crude OR (95% CI)	Adjusted OR (95% CI)^**a**^
***Paternal age***						
*< 25*	0.98 (0.92, 1.04)	1.00 (0.94, 1.07)	1.45 (1.09, 1.94)+	1.37 (1.01, 1.85)+	1.14 (1.11, 1.18)+	1.05 (1.01, 1.09)+
*25–29 (ref)*	1.00	1.00	1.00	1.00	1.00	1.00
*30–34*	1.05 (1.02, 1.09)+	1.01 (0.98, 1.05)	1.18 (0.99, 1.41)	1.18 (0.97, 1.43)	1.02 (1.00, 1.04)+	1.03 (1.01, 1.05)+
*35–39*	1.24 (1.20, 1.28)+	1.08 (1.04, 1.13)+	1.17 (0.96, 1.42)	1.10 (0.86, 1.41)	1.14 (1.12, 1.16)+	1.09 (1.06, 1.12)+
*40–44*	1.58 (1.51, 1.65)+	1.26 (1.19, 1.33)+	1.85 (1.45, 2.36)+	1.59 (1.17, 2.16)+	1.33 (1.30, 1.37)+	1.19 (1.15, 1.23)+
*45–49*	1.73 (1.60, 1.87)+	1.42 (1.31, 1.55)+	1.81 (1.17, 2.79)+	1.53 (0.94, 2.49)	1.39 (1.33, 1.46)+	1.25 (1.18, 1.32)+
*≥ 50*	1.67 (1.46, 1.90)+	1.60 (1.39, 1.84)+	2.25 (1.16, 4.39)+	2.14 (1.05, 4.38)+	1.44 (1.32, 1.57)+	1.38 (1.27, 1.51)+
***Maternal age***						
*< 20*	0.93 (0.85, 1.03)	0.89 (0.80, 0.99)+	1.31 (0.81, 2.11)	1.06 (0.65, 1.74)	1.35 (1.29, 1.42)+	1.25 (1.19, 1.32)+
*20–24*	0.89 (0.86, 0.93)+	0.89 (0.86, 0.94)+	1.07 (0.86, 1.32)	1.00 (0.79, 1.26)	1.02 (1.00, 1.04)	1.00 (0.97, 1.02)
*25–29 (ref)*	1.00	1.00	1.00	1.00	1.00	1.00
*30–34*	1.18 (1.15, 1.21)+	1.19 (1.15, 1.23)+	1.21 (1.03, 1.41)+	1.22 (1.02, 1.45)+	1.09 (1.07, 1.11)+	1.10 (1.08, 1.12)+
*35–39*	1.69 (1.63, 1.75)+	1.60 (1.53, 1.67)+	1.54 (1.26, 1.88)+	1.48 (1.14, 1.91)+	1.39 (1.36, 1.42)+	1.34 (1.30, 1.37)+
*≥ 40*	2.76 (2.58, 2.94)+	2.31 (2.14, 2.49)+	3.07 (2.18, 4.31)+	2.44 (1.64, 3.63)+	1.91 (1.82, 1.99)+	1.70 (1.62, 1.79)+
***Fetal sex***						
*Boy* vs *Girl*	0.95 (0.93, 0.97)+	0.95 (0.93, 0.97)+	0.80 (0.71, 0.91)+	0.80 (0.71, 0.91)+	1.24 (1.22, 1.25)+	1.24 (1.22, 1.25)+

*CI* confidence interval, *OR* odds ratio

^a^Adjusted for paternal age, maternal age, maternal nationality (Taiwanese or not), income (four categories) and fetal sex

+*P*-value < 0.05

With regard to eclampsia, older paternal age and maternal age were also associated with a higher risk (see Table 2 and the Fig. 1), although these effect estimates seemed less precise due to the small number of eclampsia events (prevalence 0.07%). Mothers carrying a male fetus was associated with even lower risk for eclampsia (adjusted OR: 0.80, 95% CI: 0.71, 0.91).

In the unadjusted analysis, there was a stepped increase in risk of preterm delivery as the paternal or maternal age increased (see Table 2 and the Fig. 1). For instance, infants born to mothers aged 40 or older were more likely to be a preterm delivery than infants born to mothers aged 25–29 (OR: 1.91, 95% CI: 1.82, 1.99). A higher risk was also noted among mothers < 20 years of age (OR: 1.35, 95% CI: 1.29, 1.42). After adjustment for the other parent’s age and covariates, we also noted that the effects of paternal and maternal age were attenuated. Mothers carrying a male fetus had a higher risk of preterm delivery.

The adjusted associations of paternal and maternal age with pregnancy outcomes stratified by fetal sex are presented in the Table 3 and Supplemental Figure 2. Because the number of cases for eclampsia was small, the effect estimates were not estimable. However, based on the results of gestational hypertension/preeclampsia and preterm delivery, we found that the influences of paternal and maternal age were similar in male and female fetuses.

**Table 3:** Adjusted odds ratios (95% confidence interval) for outcomes stratified by fetal sex

	Gestational hypertension or preeclampsia^**a**^		Preterm birth^**a**^	
	Boy	Girl	Boy	Girl
Total live births	704,523	643,149	704,523	643,149
Number of cases	15,585	14,941	53,052	39,611
***Paternal age***				
*< 25*	1.05 (0.97, 1.15)	0.94 (0.85, 1.03)	1.04 (0.99, 1.09)	1.07 (1.02, 1.13)+
*25–29 (ref)*	1.00	1.00	1.00	1.00
*30–34*	1.04 (0.99, 1.10)	0.98 (0.94, 1.03)	1.02 (0.99, 1.04)	1.04 (1.01, 1.07)+
*35–39*	1.10 (1.04, 1.17)+	1.08 (1.01, 1.14)+	1.07 (1.04, 1.11)+	1.11 (1.07, 1.15)+
*40–44*	1.30 (1.20, 1.40)+	1.24 (1.15, 1.34)+	1.17 (1.12, 1.22)+	1.22 (1.16, 1.28)+
*45–49*	1.42 (1.26, 1.60)+	1.44 (1.28, 1.62)+	1.22 (1.14, 1.31)+	1.29 (1.19, 1.40)+
*≥ 50*	1.55 (1.26, 1.90)+	1.67 (1.37, 2.03)+	1.29 (1.14, 1.45)+	1.51 (1.32, 1.71)+
***Maternal age***				
*< 20*	0.87 (0.75, 1.01)	0.93 (0.80, 1.08)	1.32 (1.23, 1.41)+	1.21 (1.11, 1.31)+
*20–24*	0.92 (0.86, 0.98)+	0.87 (0.82, 0.93)+	0.99 (0.96, 1.02)	1.02 (0.98, 1.05)
*25–29 (ref)*	1.00	1.00	1.00	1.00
*30–34*	1.18 (1.13, 1.23)+	1.21 (1.16, 1.27)+	1.08 (1.06, 1.11)+	1.11 (1.08, 1.14)+
*35–39*	1.54 (1.45, 1.64)+	1.68 (1.58, 1.79)+	1.33 (1.28, 1.37)+	1.35 (1.30, 1.41)+
*≥ 40*	2.21 (1.99, 2.45)+	2.46 (2.21, 2.73)+	1.63 (1.52, 1.74)+	1.80 (1.67, 1.94)+

^a^Adjusted for paternal age, maternal age, maternal nationality (Taiwanese or not), income (four categories)

+*P*-value < 0.05

A further analysis of sex ratios in all live births, births complicated by gestational hypertension/preeclampsia and births of preterm in relation to paternal and maternal age groups are shown in Table 4. In all live births, the overall sex ratio was 1.10 and increased as paternal and maternal age increased (*p* for trend < 0.001). The sex ratio for births of gestational hypertension/preeclampsia was 1.04 and for preterm deliveries was 1.34. Interestingly, we noted a tendency that as paternal and maternal ages increased, sex ratio decreased, both in births of gestational hypertension /preeclampsia and preterm, although some of these trends did not reach statistical significance.

**Table 4:** Sex ratios in different outcomes according to paternal and maternal age

	All live births		Gestational hypertension or preeclampsia		Preterm birth	
	n	Sex ratio	n	Sex ratio	n	Sex ratio
All ages	1,347,672	1.10	30,526	1.04	92,663	1.34
Paternal age, y						
< 25	75,119	1.08	1466	1.16	5604	1.31
25–29	328,463	1.09	6570	1.03	21,190	1.36
30–34	520,954	1.09	11,026	1.07	33,848	1.34
35–39	301,149	1.11	7524	1.02	21,736	1.34
40–44	93,068	1.10	2949	1.02	7753	1.31
45–49	21,974	1.11	760	0.96	1910	1.29
≥ 50	6945	1.13	231	0.89	622	1.19
	***P for trend***	***< 0.001***	***P for trend***	***0.04***	***P for trend***	***0.15***
Maternal age, y						
< 20	23,195	1.07	426	1.01	1998	1.42
20–24	185,607	1.08	3242	1.11	12,124	1.31
25–29	487,567	1.09	9545	1.05	30,696	1.37
30–34	474,148	1.10	11,010	1.04	32,143	1.33
35–39	156,352	1.12	5195	1.00	13,344	1.34
≥ 40	20,803	1.12	1108	0.98	2358	1.23
	***P for trend***	***< 0.001***	***P for trend***	***0.02***	***P for trend***	***0.20***

Note: All *p*-values were obtained from the two-sided Cochran-Armitage trend test

## Discussion

### Principal findings

These analyses were based on the Taiwanese population including 1,347,672 live birth records from 2004 to 2011. Several findings were noteworthy. First, fetal sex was associated with the pregnancy outcomes examined. There were relatively more female births from pregnancies complicated by gestational hypertension/preeclampsia or eclampsia compared to the whole population. There were relatively more males in preterm deliveries. Second, not only advanced maternal age but also advanced paternal age were associated with a higher risk for gestational hypertension/ preeclampsia, eclampsia, and preterm delivery; maternal age had a stronger effect than paternal age. Third, we did not find evidence that the effect of paternal or maternal age was dependent on fetal sex. However, we noted that in births of gestational hypertension/preeclampsia and preterm, there seemed to be increasingly more female births as the paternal and maternal age increased.

### Interpretation

Our study found that a male fetus had 24% higher risk of preterm delivery than a female fetus, which agrees with many studies [27–29]. Several explanations of the excess in males among preterm deliveries have been proposed, including that a male fetus is heavier on average than a female fetus; a male fetus is more susceptible to pregnancy complications; a male fetus is more likely to be conceived in the early fertile period [29]. By contrast, in our results, we noted a female predominance in births of gestational hypertension/preeclampsia. The research findings on the relationship between fetal sex and gestational hypertension/preeclampsia are inconsistent. Jaskolka et al. conducted a meta-analysis and found that in non-Asian samples, women carrying male fetuses had 1.05 times higher risk of preeclampsia/eclampsia than women carrying a female fetus [4]. A Japanese study observed findings similar to ours that women carrying female fetuses had a significantly higher risk of gestational hypertension/preeclampsia, in both singleton and twin pregnancies [6]. Therefore, it may be important in the future to study the role of ethnicity on the interaction between fetus and mother and how it relates to the etiology of gestational hypertension/preeclampsia.

Although the influence of paternal or maternal age on pregnancy outcomes seemed not modified by fetal sex, our analysis results on sex ratios were interesting. Despite our findings that in all live births the sex ratio increased as paternal and maternal age increased, in births of gestational hypertension/preeclampsia and preterm the sex ratio decreased when paternal or maternal age increased. Some studies have shown similar results. For instance, Rueness and colleagues analyzed 2,206,040 births in Norway and found that in complicated pregnancies such as preeclampsia the proportion of female births increased as maternal age increased [30]. Advanced maternal age may result in more adverse maternal environment, so our findings support the theory that a male fetus is more vulnerable to adverse maternal environment and is at higher risk of intrauterine death than a female fetus [7, 30]. This may also partly explain why we found less male fetuses were born to pregnancy with gestational hypertension/preeclampsia. In line with this, in some studies, a reduced sex ratio has also been observed in deliveries after war, earthquakes, and even after a collapse of the economy [31–33].

In this study, we considered jointly the influence of advanced paternal and maternal age on pregnancy outcomes, which were correlated with each other. We found that both paternal and maternal age had an independent effect on gestational hypertension/preeclampsia and on preterm delivery and that these effects seemed to be dose (age)-dependent. The current evidence on the effect of advanced paternal age is less clear than the effect of advanced maternal age. Several studies have examined the link between advanced paternal age and gestational hypertension/preeclampsia or preterm delivery, with inconsistent findings. For instance, Harlap et al. showed in the Jerusalem Perinatal Study that the risk of preeclampsia got increased as paternal age increased [34]. Researchers at Stanford University who recently analyzed over 40 million births in the United States (US) did not find an association between advanced paternal age and preeclampsia. They did, however, report that infants of fathers at an advanced age had a higher risk of preterm delivery [35].

Biologically speaking, advanced paternal age can increase the risk of adverse pregnancy outcomes. As a man ages, cumulatively there is a higher number of male germ cell divisions, more de novo point mutations, more DNA fragmentations, and more stresses to male germ cells that induce epigenetic changes, which altogether may lead to abnormal embryonic and placental growth [35]. Epidemiologic investigations have also revealed a few interesting results. For example, there is a higher risk of preeclampsia reported from mothers in relation to recent marriage, shorter duration of cohabitation with partners, use of barrier contraception, and assisted fertilization with donor sperm [34]. Hence, although the effect may be small, paternal factors do play a role in these diseases that are often considered maternal. So we suggest that more studies are needed to replicate our observations in other populations to clarify the paternal role of pregnancy outcomes.

### Strength of the study

Our study used a large nationwide population-based sample. One major strength of the study was the data linkage through the Birth Registration system in Taiwan. In Taiwan, most infants are born to married parents, so we were able to have more complete and reliable data on the biological fathers compared to some studies in Western countries. It was reported in a US study that around 17.8% of births in Ohio from 2006 to 2012 did not have paternal records [36]. Besides, our maternal disease diagnoses were defined using medical claims data from the National Health Insurance system. Most studies have relied on data recorded in the birth reporting form, which may be subject to more errors.

### Limitations of the data

Our findings were subject to residual confounding by socioeconomic status, lifestyle, comorbidities, etc. We did not have data on mother’s body mass index or parity, despite that we included both paternal and maternal age and as many covariates as we can in the regression model. Also, we did not consider factors such as subfertility and assisted reproductive technologies, as they are related to advanced paternal and maternal age and these factors may also affect pregnancy outcomes and confound our study results [37].

In our database, it was difficult to distinguish the diagnoses for gestational hypertension and preeclampsia, so we created a composite outcome after discussion with clinical experts, which may complicate the interpretation of findings. Gestational hypertension is often a provisional diagnosis for women who do not yet meet the criteria for preeclampsia. Clinicians in Taiwan mostly do not prescribe treatments for women with mild gestational hypertension so these patients would not receive the diagnostic codes. Since the prevalence of gestational hypertension/preeclampsia in our study was relatively low, we hypothesize most of our cases were with preeclampsia.

## Conclusions

The study results have implications for future etiologic research on learning the biological mechanisms that explain the association of fetal sex, maternal and paternal age with pregnancy outcomes. Association of fetal sex and gestational hypertension/preeclampsia may be dependent on race/ethnicity. Moreover, we explored whether the effect of advanced parental age would be different between male and female fetuses and it seemed that there were no discernable differences.

In a clinical setting, we emphasize the importance of considering sex dimorphism in perinatal care. These factors (fetal sex, maternal and paternal age), though simple to assess, are important for clinicians to consider who is at a higher risk of pregnancy outcomes. Besides, given the trend of delaying childbearing in Taiwan and across many countries worldwide, it is crucial to counsel couples planning their family to be aware of the potential influences of both advanced maternal and paternal age on their pregnancies.

## Supplementary information

**Additional file 1: Figure S1.** Study flow diagram. **Figure S2.** Adjusted odds ratios (ORs) for pregnancy outcomes according to paternal and maternal age categories, among male and female fetuses.

## Data Availability

Please contact Prof. Tsung Yu (tsung.yu.ncku@gmail.com) regarding the questions on availability of datasets used for the present study.

## Abbreviations

CI: Confidence interval
ICD: International Classification of Diseases
OR: Odds ratio
