# Supplementary material for: Does sex matter? Association of fetal sex and parental age with pregnancy outcomes in Taiwan: a cohort study
Source: BMC Pregnancy Childbirth. 2020 Jun 8;20:348. doi: 10.1186/s12884-020-03039-y (PMC7282132; doi:10.1186/s12884-020-03039-y)
Supplement: Supplementary file 1 — Additional file 1: Figure S1. Study flow diagram. Figure S2. Adjusted odds ratios (ORs) for pregnancy outcomes according to paternal and maternal age categories, among male and female fetuses. [file 12884_2020_3039_MOESM1_ESM.docx]

**Does Sex Matter? Association of Fetal Sex and**

**Parental Age with Pregnancy Outcomes in Taiwan: A Cohort Study**

**Tsung Yu, Ta-Sheng Chen, Fu-Wen Liang, Pao-Lin Kuo**

**Supplemental Material**

**Supplemental Figure 1. Study flow diagram**

**
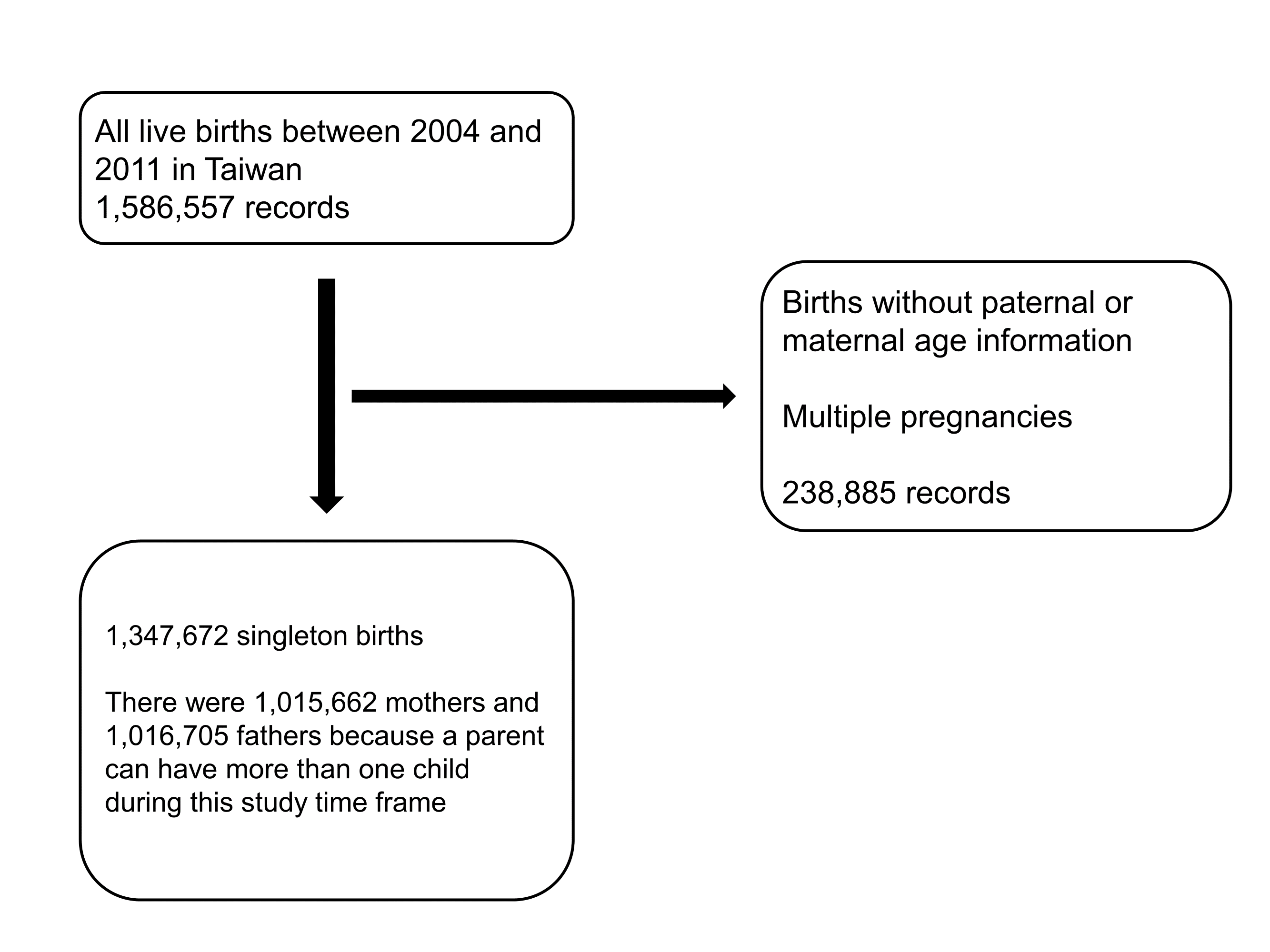
**

**Supplemental Figure 2. Adjusted odds ratios (ORs) for pregnancy outcomes according to paternal and maternal age categories, among male and female fetuses**

**
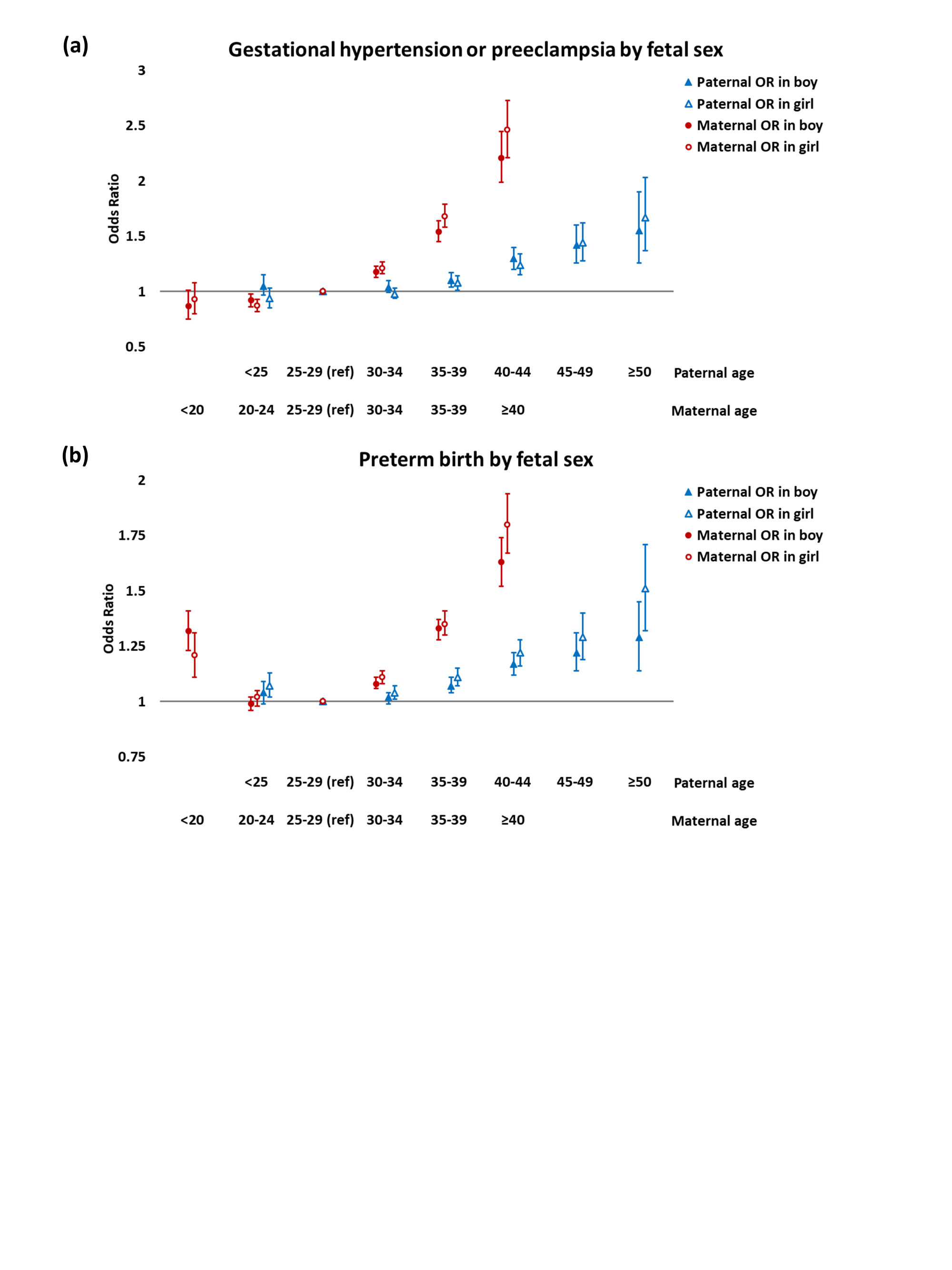
**
